# Range-Wide Genomic Analysis of Pygmy Rabbits (*Brachylagus idahoensis*) Reveals Genetic Distinctiveness of the Endangered Columbia Basin Population

**DOI:** 10.3390/genes17030335

**Published:** 2026-03-18

**Authors:** Stacey A. Nerkowski, Lisette P. Waits, Kenneth I. Warheit, Ilaria Bacchiocchi, Paul A. Hohenlohe

**Affiliations:** 1 Department of Fish and Wildlife Sciences, University of Idaho, Moscow, ID 83844, USA; stacey_nerkowski@fws.gov (S.A.N.); lwaits@uidaho.edu (L.P.W.); ilariab@uidaho.edu (I.B.); 2 U.S. Fish and Wildlife Service, Northeast Fishery Center, Lamar, PA 16848, USA; 3 Washington Department of Fish and Wildlife, Olympia, WA 98501, USA; kenneth.warheit@dfw.wa.gov; 4 Department of Biological Sciences, University of Idaho, Moscow, ID 83844, USA

**Keywords:** conservation genetics, endangered species, phylogeography, population genetic structure, RADSeq, sagebrush

## Abstract

Background/Objectives: Pygmy rabbits (*Brachylagus idahoensis*) are closely associated with sagebrush steppe habitat across the western United States, and loss and fragmentation of this habitat has contributed to the near extirpation of the Columbia Basin population in Washington state (CB). The CB pygmy rabbit was listed under the Endangered Species Act in 2003, and recovery efforts have included captive breeding, reintroduction, and genetic rescue with the translocation of rabbits from populations across the species range. Methods: We used restriction site-associated DNA sequencing (RADseq) on samples from across the species range, including CB pygmy rabbits captured prior to genetic rescue and admixture. We determined population genetic structure across the pygmy rabbit range, tested for genomic signatures of adaptive divergence among populations, assessed the genetic distinctiveness of the ancestral CB population, and identified loci useful for monitoring ancestry in the current admixed CB population. Results: Our dataset included 9794 single-nucleotide polymorphisms (SNPs) across 123 individuals. We identified four distinct genetic groups, including the central portion of the species range and three peripheral populations: CB, northern Utah/Wyoming, and southern Utah. The ancestral CB population showed the highest degree of genetic distinctiveness using multiple clustering, ordination, and genetic differentiation analyses. We identified evidence for putatively adaptive variation among populations, but no significant gene ontology associated with local adaptation. Conclusions: Our results highlight the long-term isolation of the ancestral CB population as well as historical isolation of other peripheral populations. Our results also provide SNP loci for monitoring the consequences of genetic rescue efforts in the current admixed CB population.

## 1. Introduction

Landscape structure can affect population connectivity and population size, and in turn impact the distribution of genetic diversity among populations [1,2]. These factors affect the ability of natural populations to persist and adapt to environmental change [3]. In particular, populations that inhabit the interior regions of a species’ distribution are predicted to have high connectivity and higher genetic diversity, while populations near the edge of a species’ range are often subject to fragmentation, greater isolation, more limited resources, greater habitat and environmental variability, lower genetic diversity, and higher risk of extirpation [4,5,6,7]. Here we focus on the Columbia Basin (CB) population of the pygmy rabbit (*Brachylagus idahoensis*) in Washington State, an example of an at-risk peripheral population that is the focus of intense conservation efforts.

**Figure 1:**
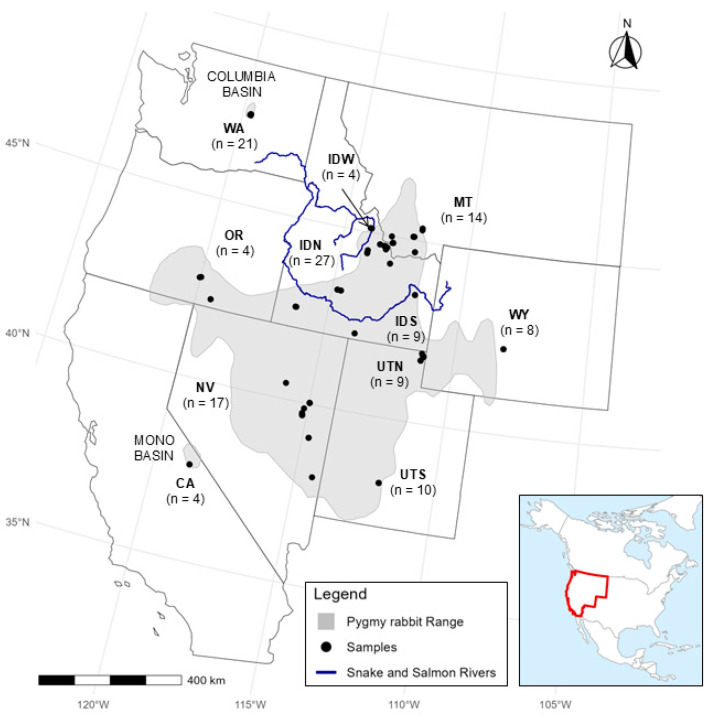
Pygmy rabbit (*Brachylagus idahoensis*) range [15] in the western United States, and sample locations for 123 pygmy rabbit samples collected from 2001 to 2018 included in the final filtered SNP dataset. Total sample sizes are represented for each region (pre-filtering sample size: post-filtering sample size), with some states split to test hypotheses about population genetic structure: California (CA, *n* = 10:4), Washington (WA, *n* = 34:21), Nevada (NV, *n* = 56:17), Oregon (OR, *n* = 6:4), Idaho north of the Snake River (IDN, *n* = 41:27), Idaho south of the Snake River (IDS, *n* = 13:5), Idaho west of the Salmon River (IDW, *n* = 4:4; these are also north of the Snake River), northern Utah (UTN, *n* = 11:9), southern Utah (UTS, *n* = 12:10), Wyoming (WY, *n* = 30:8), and Montana (MT, *n* = 15:14). Samples from WA and CA represent the isolated Columbia Basin and Mono Basin populations, respectively. Also shown are the Snake River (in southern Idaho) and the Salmon River (in central Idaho).

Pygmy rabbits are the smallest rabbit in the world and obligately associated with sagebrush (*Artemesia* species) [8,9]. They are known as the ecosystem engineers of the shrub steppe environment because they dig burrows for both temperature regulation and protection [10,11]. The historical distribution of pygmy rabbits was patchy across the sagebrush steppe habitat of the western United States, with populations in Washington, Idaho, Oregon, Utah, Nevada, Wyoming, Montana, and California (Figure 1) [9,12,13]. The distribution of sagebrush steppe has declined by over 50% due primarily to changes in land use, degradation of habitat from invasive species and wildland fire, and urban development [14]. Although the geographic extent of pygmy rabbits encompasses a fairly large area (Figure 1), their specialized habitat requirements (i.e., deep soil for burrowing) restrict them to only a small fraction of sites within their range [15]. Historically, the habitat of the Great Basin allowed for high levels of connectivity, but it has become much more fragmented over the past century [16,17,18,19]. As a result of habitat loss and population declines, the entire species was proposed for endangered status under the U.S. Endangered Species Act, but the US Fish and Wildlife Service concluded a range-wide listing was not warranted [20]. In 2023, pygmy rabbits were once again petitioned for listing, and that petition is still under consideration. With continued loss and fragmentation of sagebrush habitat [21], assessing connectivity and genetic variation among pygmy rabbit populations is important for future conservation planning [15,22].

In the northwestern portion of the species range, pygmy rabbits in the Columbia Basin of Washington state (WA-CB or CB) have been isolated for an estimated 40,000 years, based on mitochondrial DNA sequence data [23]. CB pygmy rabbits were restricted to a single population by 2001 and listed as endangered under the Endangered Species Act as a distinct population segment (DPS) in 2003 based on biogeographic separation and genetic distinctiveness [23,24,25,26]. The last 16 CB pygmy rabbits were removed from the wild in 2001 and placed into a captive breeding program. To counteract the effects of low reproductive rates and documented inbreeding depression [27], individuals from other populations (first Idaho in 2003, and several other source populations in 2011–2014 and 2020) were translocated into the CB population. The last remaining individual with pure CB ancestry died in 2006, and all individuals in the population are currently admixed [28,29]. As a result of these captive breeding and genetic rescue efforts, pregnancy rates and juvenile growth and survival increased [27], and over 2000 admixed pygmy rabbits have been released into sagebrush habitats at a cluster of sites in the central Washington recovery area [30]. However, CB ancestry represents a minority (<25%) of the current genetic composition of the admixed CB population following genetic rescue [29,31], motivating the need to better understand the genetic consequences of these recovery efforts.

Several genetic studies of pygmy rabbits have been conducted using nuclear microsatellite loci and mitochondrial cytochrome *b* for the CB population [23,29,31,32], and populations in Idaho/Montana [22,23], Wyoming [33], Oregon [23], and Nevada/California [34]. In a range-wide study using microsatellite loci, DeMay et al. [32] identified four distinct genetic groups when assessing rabbits from different regional populations: (1) CB/Idaho, (2) Nevada/Oregon, (3) northern Utah/Wyoming, and (4) southern Utah. However, this study was limited to samples from the CB population after translocation and hybridization with individuals from Idaho, so it lacked individuals representing only the ancestral CB population. A more recent genomic study [35] focused on the Great Basin and did not include any CB samples but confirmed the genetic distinctiveness of the isolated population in the Mono Basin of California.

A more complete understanding of the range-wide population genetic history and genetic differentiation among pygmy rabbits, including the distinctive CB population, requires genetic samples from the CB population prior to genetic rescue and admixture with rabbits from other states. In the current study, we were able to include historic DNA samples from preserved specimens of individuals of pure CB ancestry. Characterizing genetic variation between the ancestral CB population and other source populations can provide information for monitoring native versus introduced ancestry and understanding the genetic consequences of rescue in the currently admixed population. To this end, we also included historic DNA samples from some of the specific individuals translocated into the Columbia Basin for genetic rescue as representatives of their respective source populations.

Here, we use a reduced representation sequencing approach to identify and genotype single-nucleotide polymorphism loci (SNPs) to (1) determine population genetic structure across the pygmy rabbit species’ range, (2) assess the distinctiveness of the ancestral CB population, and (3) test for genomic signatures of adaptive divergence among populations. Across most of the species’ range, we expected low to moderate levels of genetic structure among populations, corresponding to historic landscape connectivity and distribution of habitat. We predicted that, consistent with previous microsatellite results and historical isolation prior to genetic rescue, a genome-wide set of SNPs would show the ancestral CB population to be the most genetically differentiated from other populations, whereas the Idaho populations would cluster with the Great Basin. We also expected genomic signatures of adaptive differentiation among pygmy rabbit populations, given the environmental and habitat variation across the range. Our broader goal is to provide genomic information useful for monitoring genetic diversity and the persistence of native ancestry following genetic rescue in the admixed CB population. We also seek to better understand the distribution of genetic variation and evidence for local adaptation across the species range, given ongoing conservation concerns for the species as a whole.

## 2. Materials and Methods

### 2.1. Sampling and RADseq Library Preparation

We gathered 232 tissue samples collected by researchers and managers from either (1) 2 mm ear biopsies from pygmy rabbits across the region (Figure 1) from 2001 to 2013 (*n* = 209), or (2) necropsy organ tissue samples (*n* = 23) collected from Oregon Zoo for individuals that were of pure CB ancestry (individuals taken from the wild prior to translocations, and their non-admixed offspring from the captive breeding program). These 232 samples included all 111 pygmy rabbits that were translocated into on-site breeding enclosures during reintroduction, and tissue samples from some of the last 16 CB pygmy rabbits taken from the wild in 2001. All of the CB samples represent individuals with known pure CB ancestry prior to translocations from other populations, be it wild-caught or individuals with a known pedigree in the captive population.

Samples were stored in ethanol and kept at −20 °C until a DNA extraction was performed. Genomic DNA was extracted using Qiagen Blood and Tissue extraction kit (QIAGEN, Valencia, CA, USA) following the manufacturer’s recommended protocol or through phenol-chloroform extractions. We evaluated the DNA quality of each sample using a Qubit Fluorometer (Thermo Fisher, Waltham, MA, USA) and for samples with >1.5 ng/uL, a 1.0% agarose gel electrophoresis was performed to assess DNA quality. Our RAD sequencing library preparation was performed according to the methods described in Ali et al. [36], using the restriction enzyme *Sbf-I* and without the sequence capture. Libraries were sequenced using Illumina HiSeq 4000 and Illumina NovaSeq SP 6000 (Illumina, San Diego, CA, USA) with 150 bp paired-end reads at the Vincent J. Coates Genomics Sequencing Laboratory at the University of California, Berkeley.

### 2.2. Data Filtering and SNP Genotyping

Based on the Ali et al. [36] method, the barcode and partial restriction site can occur on either the forward or reverse Illumina sequence reads. We used a custom Perl script, flip_trim_160301.pl (available at https://github.com/sakura81/PYRA_Genomics.git), to re-orient the raw sequence reads so that all reads starting at the restriction cut site were in one file, while the other reads were contained in a second file. With the software package Stacks v2.54 [37,38,39], we used process_radtags to demultiplex reads by barcode and remove reads with uncalled bases or poor sequence quality based on Phred scores less than 10. PCR duplicates were removed using the clone_filter script in Stacks.

To minimize missing data and optimize alignments, samples containing fewer than 200,000 reads were removed; read count was related to both starting DNA quantity and alignment success, and individuals with such low read counts were unlikely to pass subsequent filters on missing data. All remaining sequence reads were aligned to the European rabbit genome (*Oryctolagus cuniculus*, OryCun 2.0) using Bowtie2 v2.2.9 [40], with the following parameters: -very-sensitive, -end-to-end, -X 900. The resulting alignments were converted from sequence alignment/map format (SAM) to binary alignment map (BAM) format using samtools [41]. The ref_map.pl pipeline, in Stacks, identifies SNPs for individual samples from the reference-aligned sequence reads using a maximum likelihood approach. The ref_map.pl pipeline was run with default parameters (model marukilow and var-alpha 0.05) to create a catalog of SNPs across our sample set as a single population. To optimize parameters for calling genotypes, we used six samples for which replicates were run independently during library preparation and sequencing. We assessed the mismatch rate among the replicate pairs, using a modified version of an R script described in Mastretta-Yanes et al. [42], RAD_error_rate_K2.R (available at https://github.com/sakura81/PYRA_Genomics.git). We estimated genotype mismatch rates between the replicate pairs across 25 parameter sets, varying the minimum depth of coverage from five to nine and varying the minimum percentage of individuals genotyped at a locus from 50% to 90%. The mismatch rate for each replicate pair was calculated as the number of loci for which the genotypes were different between replicates, divided by the total number of loci types for both replicates. Data for replicate samples were then merged for inclusion in downstream analysis.

For the final set of SNPs, we used the optimized parameter values that generated the lowest genotype mismatch rates (minimum depth of coverage = 3, and minimum percent of individuals genotyped at a locus = 70%) in the populations module of STACKS. RAD loci had to be present in 70% of the individuals (-r 0.7), biallelic SNPs with minor allele frequency of at least 0.05 (-min-maf 0.05), and one SNP per RAD locus (up to 600–800 bp, covering paired-end reads) to limit linkage disequilibrium in downstream analyses (--write_single_snp and –ordered_export). This dataset was used to estimate genetic structure among the regions. Since our WA-CB samples had high levels of relatedness, we tested three levels of filtering for relatedness: (1) all individuals (no filter), (2) a pairwise relatedness maximum of 0.33, and (3) a relatedness maximum of 0.4. To determine relatedness, we used the –relatedness option in VCFTOOLS v.0.1.16 [43] which produced unbiased *ajk* values. These values were chosen on the basis of known half-sibling and full-sibling pairs and unrelated individuals. Values just under 0.33 were observed in some individuals that were in different populations, possibly resulting from ancestral admixture; thus, we tested a cutoff at 0.33 as the strictest filter, and 0.40 as a more lenient filter. The final dataset was then further filtered to remove any individuals with more than 50% missing data and to remove all SNPs in unplaced scaffolds and those located on the X chromosome using VCFTOOLS v.0.1.16.

### 2.3. Genetic Structure and Diversity

We first investigated population genetic structure using principal components analysis (PCA), using PCADAPT v4 [44]. Next, we used the program ADMIXTURE v1.3.0 [45], a maximum-likelihood clustering method. We used admixture_wrapper (https://github.com/dportik/admixture-wrapper.git, accessed on 1 January 2019) with the following parameters: a *K* = 1–20 (--kmin 1, --kmax 20), a 10-fold cross validation (–cv 10), and 50 repetitions of each *K* (--reps 50) to determine the optimal *K*. ADMIXTURE plots were visualized in the R package ggplot2 v3.4.2 [46], scatterpie v0.2.1 (https://github.com/GuangchuangYu/scatterpie.git, accessed on 1 January 2019), and ggmap v3.0.2.9001 [47]. We ran ADMIXTURE analysis on both the complete filtered set of SNPs and on a neutral set, created by removing all putatively adaptive loci (see below).

Additional population structure analyses were conducted in R package LEA v3.12.2 [48] using snmf. Snmf estimates admixture coefficients using sparse non-negative matrix factorization algorithms. Complete and neutral vcf files were input into LEA’s snmf where populations were assessed from 1 through 20 (*K* = 1:20); for diploid data (ploidy = 2), the cross-entropy criterion was used (entropy = T), the regularization parameter was set to 100 (alpha = 100), and with 50 repetitions per K value (repetitions = 50). Snmf results were visualized with ggplot2.

Once we identified the optimal number of genetic groups (*K* = 4) based on the ADMIXTURE and snmf analyses, we quantified the levels of genetic structure and diversity occurring within and between each of the groups using the R package hierfstat [49]. Vcf files for the complete and neutral sets were imported in R and populations were assigned to one of the four genetic groups identified. Observed and expected heterozygosity levels were determined using the basic.stats parameter of hierfstat, pairwise *F*_st_ using genet.dist (method = WC84), and private alleles in the R package poppr [50,51].

### 2.4. Adaptive Loci and Gene Ontology

We used three different programs to identify outlier loci with signatures of divergent selection between the genetic clusters identified above. The first was the R package pcadapt v4 [44], which uses PCA and Mahalanobis distance to provide a better ranking of candidate SNPs under local selection pressures. Outlier loci were determined by using the pcadapt program with the optimal genetic groups. We set the false discovery rate α to 0.05 and applied Benjamini and Hochberg adjustment methods [52] (method = “BH”). Results were visualized using the R package qqman [53].

Second, we used the R package OutFLANK v0.2 [54] that infers the distribution of *F*_ST_ for neutral markers assigning q-values to each locus to detect outliers that may be due to spatially heterogenous selection. The complete vcf file was imported into R using R package vcfR v1.14.0 [55] and run in OutFLANK using the following parameters: LeftTrimFraction = 0.01, RightTrimFraction = 0.01, qthreshold (false discovery rate) = 0.05, Hmin (minimum heterozygosity required) = 0.05, and NumberofSamples (number of spatial groups) = 1–9. Individuals were assigned to populations for each *K* = 1–9 as determined from ADMIXTURE, as described above. Additionally, we tested populations grouped by U.S. state, with an additional split between northern and southern Utah, resulting in 9 populations.

Lastly, we used the *F*_ST_-outlier approach implemented in BayeScan [56]. Standard PLINK files were converted to BayeScan format with PGDSpider v2.0.7. 3 [57]. The analyses consisted of 20 pilot runs of 5000 iterations, a burn-in of 50,000 iterations, a thinning interval of 10 (5000 iterations were used for the estimation of posterior odds) resulting in a total number of 100,000 iterations, and a different prior odds ratio of 100, 1000, and 10,000 (prior belief that a selection model is 1/100, 1/1000, and 1/10,000 as likely as the neutral model for a given SNP). As above, populations were assigned based on *K* = 1–9 genetic clusters and also by state. Overlapping loci among the three methods were assessed with R package ggvendiagram v1.2 [58]. To create a putatively neutral set of SNPs for further analyses, we conservatively removed loci identified as outliers by any of the three methods; both the complete and the neutral SNP set were used in the population structure analyses described above.

To gain insight into the ecological and biological functions of putative adaptive loci in pygmy rabbits, we identified candidate loci found within the genes and the Gene Ontology (GO) terms associated with such genes [59]. We used the outlier loci identified in BayeScan, OutFLANK, and pcadapt and the R program SNP2GO [60] to identify cellular component, biological process, and molecular function GO terms associated with the candidate loci using an FDR of 0.05, and following the annotations of the European rabbit genome (*O. cuniculus*, OryCun 2.0). We tested for enrichment considering all the candidates identified by pcadapt and BayeScan analyses combined (OutFLANK did not identify any adaptive loci), as well as for each analysis individually.

## 3. Results

### 3.1. RADseq Data and SNP Datasets

We obtained a total of 594,124,719 read pairs across 232 individuals, representing the regional pygmy rabbit populations. Ninety-three samples were removed from the dataset because they contained fewer than 200,000 reads, likely a result of lower starting DNA quality from historical samples. Average alignment (consisting of unique alignments of pairs averaged across individuals) to the European rabbit genome was 62%. The initial catalog created in Stacks contained a total of 132,932 putative loci. Filtering for depth of coverage, missing data, linkage, and minor allele frequency left 12,084 biallelic SNPs in a total of 123 individuals (Figure 1; Supplemental Table S1). After further removing any SNPs in unplaced scaffolds (*n* = 2103) or on the X chromosome (*n* = 186), a total of 9794 SNPs comprised the final complete set for further analysis. For the complete set, missing data per individual ranged from 2.2% to 49.5% (mean 17.7%), missing data per SNP ranged from 0.8% to 30.0% (mean 17.7%), mean depth per individual ranged from 2.7 to 47.2 (mean 9.3), and mean depth per site ranged from 2.8 to 29.3 (mean 9.9). The neutral set of SNPs excluded all putatively adaptive loci (see details on adaptive loci below), resulting in 8959 SNPs. For the neutral set, missing data per individual ranged from 2.3% to 49.9% (mean 17.9%), missing data per SNP ranged from 2.4% to 30.0% (mean 17.9%), mean depth per individual ranged from 2.8 to 47.5 (mean 9.3), and mean depth per site ranged from 3.4 to 29.3 (mean 10.0).

### 3.2. Genetic Structure and Diversity

**Figure 2:**
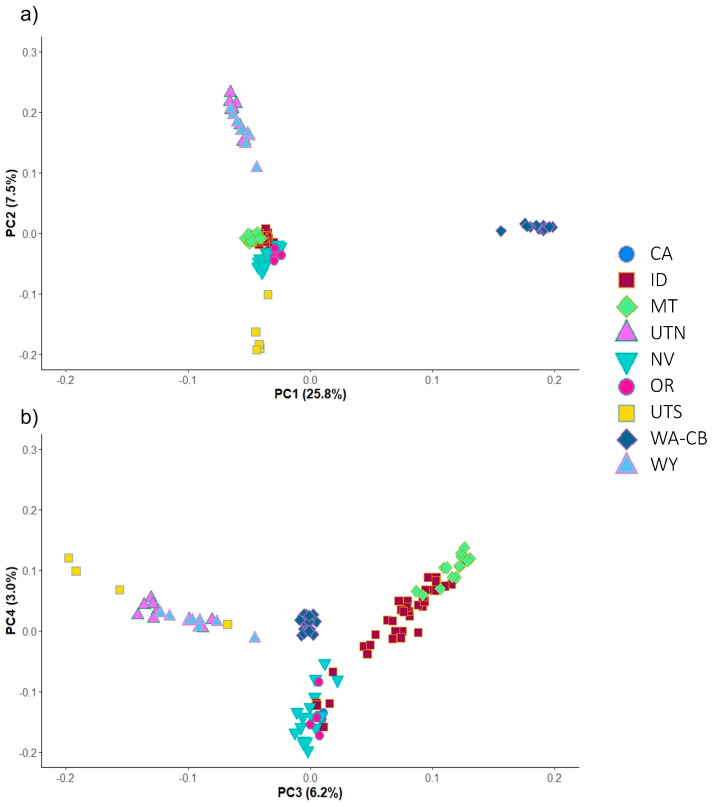
Principal components analysis (pcadapt) on the complete dataset (9794 SNPs) of 123 individuals color-coded by state (CA: California; ID: Idaho; MT: Montana; NV: Nevada; OR: Oregon; UTN: northern Utah; UTS: southern Utah; WA: Washington; WY: Wyoming), with percent variance explained on each axis in parentheses. Note that the CA samples are largely hidden behind the NV samples in both plots. (**a**) Principal components 1 and 2. (**b**) Principal components 3 and 4.

Overall, genetic clustering of the complete and neutral data sets containing 123 individuals with ADMIXTURE, *snmf*, and PCA all supported the presence of four major groups across the pygmy rabbit range, and results were similar for the complete and neutral datasets. In PCA (Figure 2), the CB population is differentiated from the other regions along PC axis 1, which explains 25% of the variation. The remaining three groups are differentiated along axis 2, including northern Utah (UTN) and Wyoming (WY) clustering together, southern UT (UTS), and the remaining samples representing the central portion of the species range. Variation within this central group is captured along axes 3 and 4 (Figure 2).

**Figure 3:**
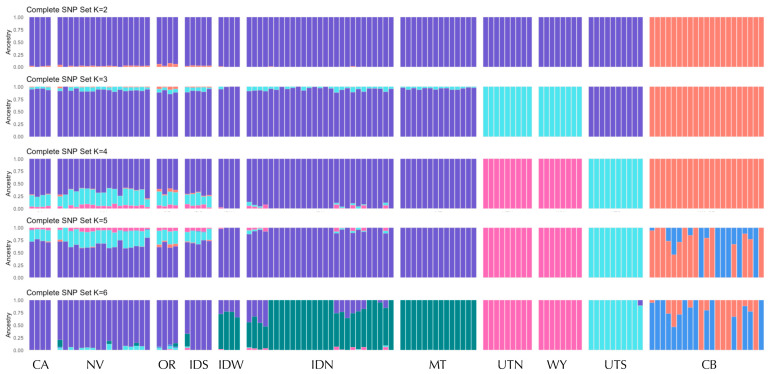
Ancestry assignments (ADMIXTURE) for 123 pygmy rabbit samples genotyped at 9794 SNPs (complete set) for *K* = 2–6. *K* = 4 is the optimal value based on lowest cross-validation values (Figure S1). Samples are ordered north–south within each region. Regions and samples sizes are as shown in Figure 1.

For the ADMIXTURE analyses, the cross-validation (CV) values from ADMIXTURE minimized at *K* = 4 (Figure S1) for both neutral and complete data sets, which showed very similar results (Figure 3 and Figure S2). At *K* = 2, CB consistently split from all other populations, and in most replicates UTN and WY separated as a group at *K* = 3. At *K* = 4, four distinct genetic groups were consistently identified: (1) CB, (2) UTS, (3) UTN and WY, and (4) central range (includes populations from Nevada (NV), Oregon (OR), Idaho (ID), Montana (MT), and California (CA)). These results through *K* = 4 are very similar when close relatives are removed from the CB population (Figure S3). At *K* = 5, the CB cluster is split into two groups representing known close relatives based on pedigree information in the captive breeding program when all individuals are included (Figure 3). When close relatives are removed, this split within CB is not observed, and instead further genetic clustering within the central group is observed at *K* = 5 and *K* = 6 (Figure S3). The results of *snmf* were generally similar to those of ADMIXTURE, with the optimal number of groups at *K* = 4 (Figure S1), at which the same four genetic clusters are identified for both the complete and neutral SNP sets (Figure S4).

**Figure 4:**
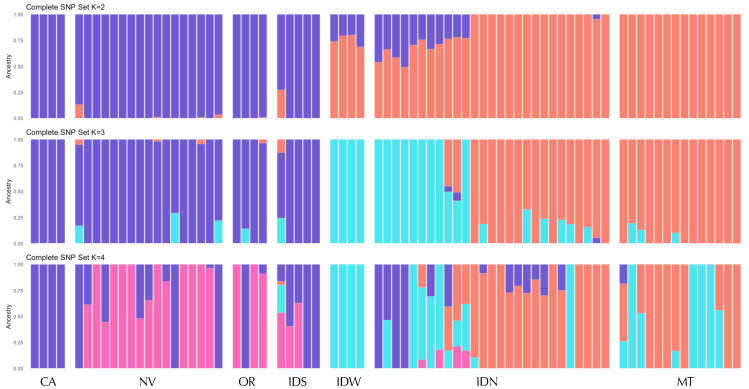
Ancestry assignments (ADMIXTURE) for 75 individuals within the central portion of the species range genotyped at 9794 SNPs (complete set) for *K* = 2–4. Samples are ordered north–south within each region. Regions and samples sizes are as shown in Figure 1.

Within the central range group, we found evidence for further genetic structure, including differentiation between southern (NV, OR, CA, and ID south of the Snake River) and northern (all samples north of the Snake River: IDN, IDW, and MT) regions. This southern cluster mostly corresponds to the Great Basin (GB; Figure 1), so we refer to this as the GB cluster (although note that some geographic depictions of the GB extend to the southwest corner of WY and would cover part of the UTN-WY genetic cluster). In the ADMIXTURE results for the complete dataset, there is some admixture or genetic similarity between the GB cluster and UTS at *K* = 4, and a split between the IDN-MT and GB clusters at *K* = 6 (Figure 3 and Figure S2). To investigate these patterns further, we conducted ADMIXTURE and snmf for the subset of 75 individuals from within the central range region. While cross-validation and entropy analyses suggested a single cluster (Figure S5), at *K* = 2, we observed the same split of northern and southern groups, separated by the Snake River (Figure 4). At higher K values of 3 and 4, we observed evidence for genetic differentiation within ID, including in samples from north of the Snake River and west of the Salmon River (IDW; see Figure 1). Additionally, the isolated Mono Basin population in CA showed some evidence of genetic distinctiveness. Results were largely similar for the complete and neutral SNP sets across ADMIXTURE and snmf (Figures S5–S7).

We found moderate differences in genetic diversity among the four genetic groups identified (Table 1). Examining polymorphic loci only, nucleotide diversity (π) ranged from 0.07 to 0.15, in which the CB and UTS populations had the lowest diversity (π = 0.07). The values were nearly identical between the complete and neutral data sets. Observed heterozygosity levels were lowest in CB (H_o_ = 0.10) compared to the central range (H_o_ = 0.15), UTN-WY and UTS (H_o_ = 0.14) groups, with minimal differences between the complete and neutral data sets (Table 1). We also identified private alleles within each group, of which 11–25% were adaptive, with the central range having the most (Table 1). These results were qualitatively similar when close relatives were removed within the CB population (Table S2). These results are consistent with the conclusion that the central range group contains the greatest genetic diversity in pygmy rabbits, consistent with its large geographic extent leading to a relatively larger historic effective population size. Observed heterozygosity was lower than expected in all groups, consistent with inbreeding and/or the Wahlund effect across structured populations.

Pairwise *F*_ST_ values among the four defined genetic groups were nearly identical between the two SNP datasets with values varying between 0.12 and 0.53 (Table 2) in both sets. Pairwise *F*_ST_ values reflected the greatest divergence between the CB group and UTS and UTN-WY (*F*_ST_ = 0.52 and 0.53, respectively), reflecting the isolation of these peripheral populations. These results also held when close relatives were removed in the CB population (Table S2).

### 3.3. Adaptive Loci and Gene Ontology

**Figure 5:**
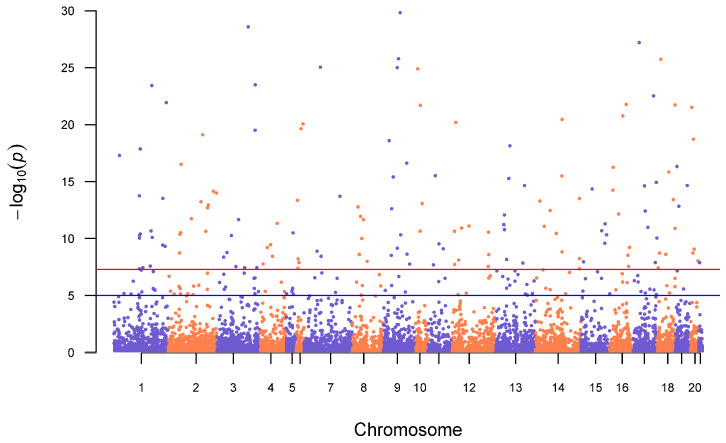
Outlier loci identified by pcadapt across 9794 SNPs (complete set) genotyped in 123 individuals. Red line represents genome-wide significance threshold of *p* = 5.00 × 10^−8^, while the blue line corresponds to the suggestive threshold of *p* = 1.00 × 10^−5^. Alternating blue and orange colors represent chromosomes, using alignment to the European rabbit reference genome assembly.

A total of 827 putatively adaptive SNPs were identified in pcadapt, using the four genetic clusters described above. We identified 18 SNPs in BayeScan in which populations were assigned by states, and 23 SNPs in which populations were assigned by region at *K* = 9. The SNPs found in BayeScan at lower K values up to 9 are fully represented in the 18 and 23 SNPs. OutFLANK identified no outlier SNPs. When assessing the overlap between the three analyses, 807 (97%) were unique to pcadapt, two in the state-assigned BayeScan run, and five in the regionally assigned BayeScan run; most loci identified by BayeScan were also identified by pcadapt (Figure S8). Of the 827 putatively adaptive SNPs, 386 (46.7%) were found within genes annotated in the European rabbit reference genome. Despite the identification of candidate loci under selection among portions of the species’ range, no significant GO term enrichment was identified using SNP2GO. Additionally, the outlier loci identified by pcadapt does not fall in one region of a chromosome but are scattered across the genome (Figure 5), suggesting that there is no single major-effect locus contributing to adaptive differentiation.

## 4. Discussion

### 4.1. Genetic Structure and Evolutionary History

**Figure 6:**
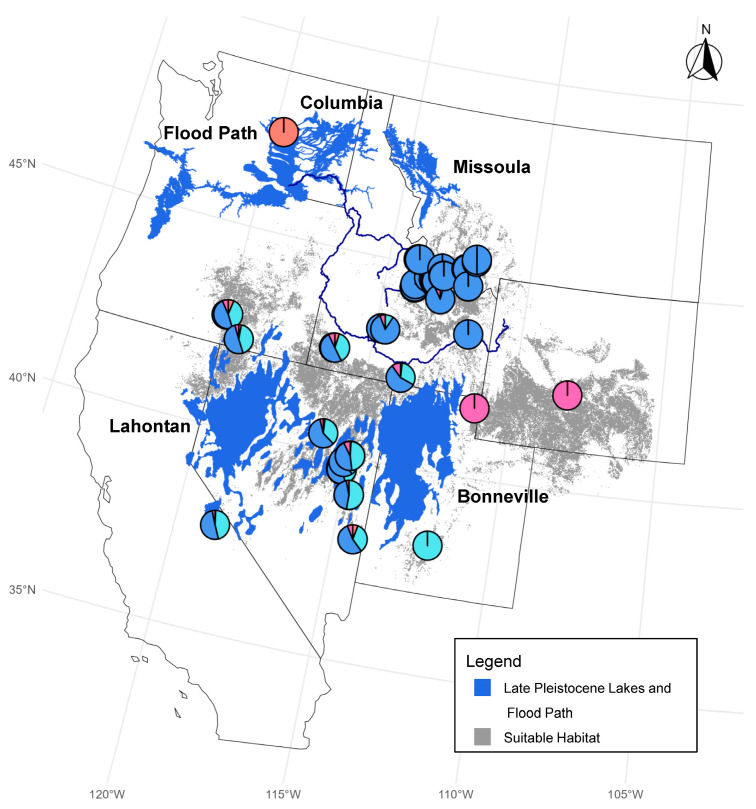
Suitable habitat and historic landscape features affecting population genetic structure in pygmy rabbits. Shown are the locations of glacial Lake Missoula, glacial Lake Columbia, pluvial Lake Bonneville, and pluvial Lake Lahontan during the Late Pleistocene. The flood path includes the areas affected by periodic flooding by outflows from glacial Lake Missoula during the Pleistocene. Suitable habitat for pygmy rabbits is based on [15]. Pie charts represent proportional genetic ancestry at each sampling location for *K* = 4, as shown in Figure 3.

This study offers insight into the potential historical groups and conservation units of pygmy rabbits across the western United States, including peripheral populations such as the endangered population within the Columbia Basin of Washington state (CB). Here, we conducted a genome-wide survey of the genetic variation in pygmy rabbits, using RADseq to provide the first range-wide dataset of SNPs for this vulnerable species. By using historical DNA samples collected prior to recent translocations from other populations for genetic rescue, we were able to characterize the genetic composition of the ancestral CB population. Our data suggest that four main evolutionary lineages occur across the pygmy rabbit range: (1) CB, (2) northern Utah and Wyoming, (3) southern Utah, and (4) the central portion of the species range (California, Nevada, Oregon, Idaho, and Montana) (Figure 1 and Figure 6). These results confirm the findings of Warheit [23], who used nine microsatellite loci and mitochondrial DNA sequence data to quantify the genetic distinctiveness of the Columbia Basin population compared to populations in Idaho, Oregon, and Montana. Our results are also consistent with those of DeMay et al. [31]; however, that study was unable to separate Columbia Basin and Idaho ancestry due to the lack of pure (non-admixed) Columbia Basin samples in their dataset. Our estimates of high genetic differentiation (*F*_ST_) between the ancestral CB population and other groups reinforce the finding of distinctiveness that warranted the CB pygmy rabbit its initial protection as an endangered distinct population segment (DPS) under the Endangered Species Act in 2003. The current admixed population has maintained some original CB ancestry, but it contains substantial genetic variation from other portions of the species’ range [29].

The pronounced genetic differentiation of the CB population is consistent with patterns of genetic divergence observed among diverse taxa in the Pacific and Inland northwest regions of Washington state [61], where Pleistocene glacial refugia and vicariance are hypothesized to have shaped genetic structure for co-distributed species [62,63]. The Columbia Basin in Washington contains a sagebrush steppe habitat that is largely surrounded by mesic coniferous forests that represent significant barriers to gene flow and dispersal for sagebrush-obligate species [64], and likely contributes to the elevated degree of distinctiveness observed in this study and in other sagebrush-obligate species in this region (e.g., [61,65]). Oh et al. [65] suggested that increasing geographic isolation and restriction of gene flow, accompanied by declines in effective population sizes during the last glacial period, led to the divergence in other sagebrush-obligate species, the Greater sage-grouse (*Centrocercus urophasianus*) and Gunnison sage-grouse (*C. minimus*). Population differentiation by genetic drift and substantial range contraction during the late Pleistocene has been inferred for many bird species in the region [65,66], and it is consistent with the patterns of genetic structure observed here.

In addition to CB, the levels of genetic differentiation (*F*_ST_) between the central range, southern Utah, northern Utah, and Wyoming pygmy rabbit populations demonstrated other peripheral populations across the species range. Historically, these populations may have had a greater level of connectivity [35], but likely became more isolated recently due to loss of habitat and fragmentation in the sagebrush steppe ecosystem [67]. Grayson [67] suggested that pygmy rabbit populations began declining at the end of the Pleistocene, reducing the connectivity of the populations ~10,000 years ago. The Mono Basin population of pygmy rabbits, the only portion of California in which the species occurs, has also been identified as a genetically distinct population [34,35]. These previous studies used microsatellites [34] and RADseq [35] to assess genetic population structure and adaptive divergence among pygmy rabbits in the Mono Basin, Nevada, and southern Oregon, roughly covering the southwestern half of what we identified as the central range cluster. In our study, the Mono Basin population did not separate as a distinct genetic group when comparing populations across the pygmy rabbit range up through *K* = 6. Furthermore, it did not form a distinct genetic group among just the central range samples until *K* = 4 (Figure 4), suggesting that cessation of gene flow between the Nevada and California populations is more recent than among other populations across the species range. However, it is worth noting that our sample size of Mono Basin individuals was quite small (*n* = 4), such that genetic variants unique to this population could have been lost during filtering for minor allele frequency. Thus, our analysis may underestimate the genetic distinctiveness of the Mono Basin pygmy rabbit population. Byer et al. [35] found evidence that the California population split from the rest of the southwestern Great Basin 64,000–214,000 YBP according to coalescent simulations, and that it exhibits adaptive differentiation. This timing is much older than the estimated split of the Columbia Basin population from the central populations, ~40,000 years ago using a mtDNA molecular clock approach [23]. Further assessment of divergence timing among pygmy rabbit genetic groups, including the Mono Basin population, would be valuable.

Within the largest of the four major genetic regions identified across the species range, we found strong evidence for further genetic structuring in the central portion. This was apparent in the range-wide analysis (e.g., support for *K* = 6 in the *snmf* cross-entropy results; Figure S1) as well as separate analysis of the central range subset. Within the central range, at *K* = 2 the Idaho populations north of the Snake River and Montana samples separated from the remaining regional populations (Nevada, Oregon, and California), which are largely within the Great Basin (Figure 1). Our results suggest that environmental factors near the Snake River, or the river itself, may have acted as a major barrier to gene flow and dispersal over the recent evolutionary history of pygmy rabbits. Estes-Zumpf and Rachlow [68] demonstrated how landscape features such as rivers, creeks and roads can act as filters or barriers to dispersal in pygmy rabbits. They documented pygmy rabbits crossing perennial streams, but the occurrences were rare. Additionally, the IDW samples located west of the Salmon River were a somewhat distinct group from the other IDN and MT samples at *K* = 4, suggesting rivers may act as a semi-permeable barrier to gene flow in pygmy rabbit populations.

The current distribution of pygmy rabbits is highly patchy within their geographic range. Based on the spatial distribution of the four genetic groups, we hypothesize that Pleistocene lakes (Missoula, Bonneville, and Lahontan) may have played a role in limiting historic dispersal and gene flow among portions of the pygmy rabbit range, contributing to the evolutionary lineages identified in this study. Pleistocene lakes and glaciers covered much of the Pacific Northwest and Great Basin regions (Figure 6), limiting dispersal of plants and animals [15,69]. Some lake floors, such as that of glacial Lake Missoula, were rapidly colonized by grasses and sagebrush after the lakes contracted, whereas pluvial lake Bonneville remained sparsely vegetated [69]. Current gaps in habitat in northern to central Utah and western Nevada coincide with Pleistocene lakes Bonneville and Lahontan [15]. The presence of Pleistocene lakes, and also their draining, could result in persistent limits to dispersal or suitable habitat for pygmy rabbits across Utah. This could explain the distinct genetic northern Utah/Wyoming and southern Utah genetic groups identified here.

However, other evidence supports the role of sagebrush habitat distribution, rather than Pleistocene lakes, in driving some of the population genetic structure we observed across the pygmy rabbit range. Byer et al. [35] specifically tested these alternatives in a landscape genetic study and found that historic sagebrush distribution provided more explanatory power than pluvial lakes for the Great Basin portion of the pygmy rabbit range. In our study, the genetic distinctiveness of the CB population likely cannot be explained by the geographic location of Pleistocene Lake Missoula, which lay to the northeast of the current pygmy rabbit range (Figure 6). Periodic outflow floods from Lake Missoula during the Pleistocene would have impacted pygmy rabbit populations in the Columbia Basin, perhaps causing population bottlenecks and loss of genetic variation, but the timing of isolation of this population likely predates the Lake Missoula floods [23]. It is more likely that long-term lack of connectivity in sagebrush habitat between the Columbia Basin in Washington and the rest of the species range, including neighboring populations in Oregon and Idaho, accounts for the historic isolation and genetic differentiation of CB pygmy rabbits.

### 4.2. Genetic Diversity and Adaptive Variation

The CB population of pygmy rabbits showed lower levels of genetic diversity than the other genetic groups. The low level of diversity within this population is likely the result of both long-term isolation and the more recent (1990s) severe population bottleneck. Research conducted on samples collected before this period documented higher levels of genetic diversity and lower pairwise *F*_ST_ levels for historic specimens collected from 1948 to 1979 [23]. Reduction in nucleotide diversity was also identified in the CB population of greater sage grouse and was attributed to similar factors of long-term isolation and recent habitat loss [65]. The captive breeding program was originally designed to prevent the extirpation of the Washington pygmy rabbit population, but was hampered by the low genetic diversity and low population growth rates. The program suffered from low pregnancy success, low juvenile growth, and low juvenile survival, suggesting inbreeding depression within the captive population [27]. Genetic rescue using individuals from Idaho populations increased genetic diversity in the breeding program, and higher pregnancy rates, juvenile growth, and juvenile survival were documented [27,29].

Isolated populations have an increased tendency to lose genetic variation, which increases the risk of extinction due to a reduced ability to adapt to environmental change [70,71,72]. Compared to populations near the core, populations at the edges of geographic ranges may experience reductions in effective population size and genetic diversity, creating increased genetic differentiation [6,73]. The central range group exhibited the highest levels of genetic diversity in our study, with both heterozygosity and number of private alleles far exceeding the CB population, which is congruent with other studies done in Idaho and Nevada [22,34]. Likewise, microsatellite studies showed reductions in genetic diversity with increasing distance from the core of the geographic range for Wyoming and Idaho populations [33,74]. The central portion of the pygmy rabbit range has likely had a greater geographic extent of contiguous sagebrush habitat and a larger effective population size of pygmy rabbits, compared to more isolated, peripheral populations such as CB, northern UT/WY, southern UT, and the Mono Basin.

Lack of connectivity can favor local adaptation by reducing the homogenizing effects of gene flow [75]. Our analysis showed a lack of connectivity among the four genetic groups across the pygmy rabbit range and identified outlier loci exhibiting signatures of divergent selection among populations. We did not find any gene ontology terms that were significantly enriched within our set of putative adaptive loci, and outlier SNP loci were scattered across the genome. This is consistent with local adaptations among pygmy rabbit populations being polygenic and linked to multiple phenotypic traits. However, it may also reflect a lack of statistical power to detect adaptive variation given our sample sizes, elevated levels of genome-wide differentiation among population groups, or the reduced representation nature of our RADseq approach that may not capture large portions of genomic variation. However, it is likely that pygmy rabbit populations, like other sagebrush-obligate species, are adapted to local varieties of sagebrush, which produces high concentrations and diversity of plant secondary metabolites that can be challenging for herbivory [35,65]. Signatures of local adaptation in pygmy rabbit populations across the southern Great Basin have been shown to be partially driven by variation in sagebrush, specifically linked to gene regions associated with metal ion-binding proteins that are involved in multiple metabolic pathways including binding of toxic compounds [35]. Similarly, in a whole-genome sequencing study Oh et al. [65] found locally adaptive loci in sage-grouse that were responsible for dietary adaptation and detoxification of plant secondary metabolites. Chemical variation in sagebrush across the pygmy rabbit range likely plays a role in local adaptation and may possibly limit dispersal among pygmy rabbit populations.

### 4.3. Conservation Implications

Identifying distinct genetic groups across the pygmy rabbit range using thousands of SNP loci can help guide future management actions for pygmy rabbits, especially the endangered, reintroduced, and admixed population within the Columbia Basin. SNPs that we identified here could be used to design panels of markers for high-throughput genotyping techniques such as GT-Seq [76] or SNP chip arrays [77] for long-term genetic monitoring to assess individual identity, ancestry, parentage, and adaptive variation. Our increased understanding of genetic diversity and divergence across the range could help guide management strategies for additional augmentation into the CB population. Since the central range had the greatest amount of genetic diversity and the lowest divergence values to CB, this information already has been used by managers to prioritize trapping sites for augmentation as part of demographic rescue in March 2020. Similar to the extent that pygmy rabbits are locally adapted to different sagebrush habitats across their range, source populations from the northern part of the central range are also expected to be most similar to CB, but further genomic study of adaptive variation could inform the specific choice of source populations.

Additionally, the reintroduced, admixed population in Washington provides a unique opportunity to track the impact of selection on genetic variation drawn from multiple pygmy rabbit populations from divergent evolutionary lineages, in the context of a largely successful genetic rescue of a critically endangered population [29]. Using a panel of markers from the loci we have identified here, including those we identified as private alleles and others with large allele frequency differences that could be diagnostic, it would be possible to track ancestry from the different source populations across chromosomal regions and potentially identify candidate genes responsible for persistent CB ancestry, and/or adaptive introgression of variation from other populations that is favored in the Columbia Basin environment. If ancestral Columbia Basin ancestry is favored by selection in the admixed population, our results predict that these variants are likely to be spread across the genome on multiple chromosomes, based on the patterns of local adaptation we found.

Finally, our results reinforce the genetic distinctiveness of not only the Columbia Basin, but also other peripheral populations of pygmy rabbits, including northern Utah/Wyoming, southern Utah, and the Mono Basin of California, that may reflect historical isolation from the core species range. Each of these populations exhibits genetic diversity roughly intermediate between the Columbia Basin and central range groups. While we did not attempt to formally delineate conservation units for the species in this study, our results provide an outline of population groups based on genomic-scale data. Further genetic and demographic work in each of these areas, including monitoring, would be valuable for measuring population trends, and data on habitat or other factors within each area could inform assessments of conservation status for pygmy rabbits.

## Figures and Tables

**Table 1:** Genetic diversity statistics for pygmy rabbit populations in the central range (CR), Washington Columbia Basin (CB), northern Utah and Wyoming (UTN-WY), and southern Utah (UTS) genetic groups. Statistics were calculated in STACKS version 2.54 for the complete data set (9794 SNPs) and neutral data set (8959 SNPs) using population assignments at *K* = 4 (Figure 2).

	Complete Set				Neutral Set			
	Populations							
Genetic Diversity Statistics	CR	CB	UTN-WY	UTS	CR	CB	UTN-WY	UTS
Total Individuals	75	21	17	10	75	21	17	10
Mean Genotyped Individuals per Locus	39.9	12.3	10.3	6.8	40.2	12.2	10.4	6.8
Observed Heterozygosity	0.14	0.10	0.15	0.15	0.15	0.10	0.15	0.15
Expected Heterozygosity	0.20	0.12	0.18	0.18	0.21	0.13	0.19	0.18
Private Alleles	53	11	6	4	47	9	5	3
% of Private Alleles that were Putatively Adaptive	11.3%	18.2%	16.7%	25.0%	-	-	-	-

**Table 2:** Pairwise genetic differentiation (*F*_ST_) for the complete data set (above diagonal, 9794 single-nucleotide polymorphisms (SNPs)) and *F*_ST_ for the neutral data set (below diagonal, 8959 SNPs) for each of the four genetic groups identified as best-supported in clustering analyses: Washington Columbia Basin (CB), central range, northern Utah and Wyoming (UTN-WY), and southern Utah (UTS).

	CB	Central Range	UTN-WY	UTS
CB	-	0.40	0.52	0.53
Central range	0.31	-	0.13	0.16
UTN-WY	0.44	0.12	-	0.28
UTS	0.45	0.12	0.24	-

## Data Availability

Custom scripts and input files used for data analysis are available at https://github.com/sakura81/PYRA_Genomics.git. Raw RADseq data is available on the NCBI SRA (BioProject PRJNA1430080), and a metadata file and a vcf file of the complete SNP data set are available at the Dryad Digital Repository (doi: 10.5061/dryad.t1g1jwth8).

## Supplementary Materials

The following supporting information can be downloaded at: https://www.mdpi.com/article/10.3390/genes17030335/s1, Table S1: Individual identification, sampling location, latitude/longitude, and region (as shown in Figure 1) for 123 samples included in the final filtered dataset; Table S2: (a) FST values and (b) Heterozygosity levels in pygmy rabbit samples genotyped at 9794 SNPs (complete set) with relatedness cutoff values of >0.33 and >0.40 for Washington samples; Figure S1: ADMIXTURE cross-validation error plots for *K* = 1–20 across 123 pygmy rabbit samples genotyped at (a) 9794 SNPs (complete set) and (b) 8958 SNPs (neutral set); Figures S2 and S3: Graphical representation of ancestry assignments from ADMIXTURE; Figure S4: Graphical representation of ancestry assignments from snmf for 123 pygmy rabbit samples; Figure S5: ADMIXTURE cross-validation error plots for 50 replicates of *K* = 1–20 across 75 pygmy rabbit samples; Figures S6 and S7: Graphical representation of ancestry assignments from ADMIXTURE for 75 central-range pygmy rabbit samples; Figure S8: Venn Diagram representing the outlier loci identified with pcadapt and BayeScan on the complete pygmy rabbit data set consisting of 9794 SNPs across 123 samples.

## Abbreviations

The following abbreviations are used in this manuscript:

RADseq	Restriction-site-Associated DNA sequencing
CB	Columbia Basin (here referring to the portion within Washington state)
CA	California
ID	Idaho
IDN	Idaho, north of the Snake River
IDS	Idaho, south of the Snake River
IDW	Idaho, west of the Salmon River (and also north of the Snake River)
MT	Montana
NV	Nevada
OR	Oregon
UT	Utah
UTN	Utah, northern (close to Wyoming border)
UTS	Utah, southern
WA	Washington
SNPs	Single-nucleotide polymorphisms
