# Supplementary material for: Range-Wide Genomic Analysis of Pygmy Rabbits (Brachylagus idahoensis) Reveals Genetic Distinctiveness of the Endangered Columbia Basin Population
Source: Genes (Basel). 2026 Mar 18;17(3):335. doi: 10.3390/genes17030335 (PMC13025606; doi:10.3390/genes17030335)
Supplement: Supplementary file 1 [file genes-17-00335-s001.zip › genes-4154416-supplementary.pdf]

Table S1: Individual identification, sampling location, latitude/longitude, and region (as shown in Figure 1) for 123 samples included in the final filtered dataset.

Individual	Location	Lat	Long	State	Abbreviation
KCLA-B01	MonoBsn	37.650756	-118.72867	California	CA
KCLA-B03	MonoBsn	37.650756	-118.72867	California	CA
KCLA-B04	MonoBsn	37.650756	-118.72867	California	CA
KCLA-B16	MonoBsn	37.650756	-118.72867	California	CA
ARANEA	ColumbBsn	47.470159	-119.69782	Washington	WA-CB
BASIL	ColumbBsn	47.491979	-119.67059	Washington	WA-CB
BESS	ColumbBsn	47.4975	-119.67977	Washington	WA-CB
BRYN	ColumbBsn	47.472053	-119.69673	Washington	WA-CB
CHAEN	ColumbBsn	47.47931	-119.69634	Washington	WA-CB
CHIVE	ColumbBsn	47.486249	-119.67487	Washington	WA-CB
COMET	ColumbBsn	47.469582	-119.68717	Washington	WA-CB
ELYMUS	ColumbBsn	47.465901	-119.6783	Washington	WA-CB
EMMET	ColumbBsn	47.463007	-119.66764	Washington	WA-CB
ESTHER	ColumbBsn	47.494818	-119.66515	Washington	WA-CB
FERN	ColumbBsn	47.481782	-119.67767	Washington	WA-CB
LOLO	ColumbBsn	47.474104	-119.67215	Washington	WA-CB
LUKEY	ColumbBsn	47.477364	-119.66546	Washington	WA-CB
MICHAELANGELO	ColumbBsn	47.489772	-119.6818	Washington	WA-CB
MYRTLE	ColumbBsn	47.495818	-119.6744	Washington	WA-CB
OTHELLO	ColumbBsn	47.471791	-119.68312	Washington	WA-CB
PEAT	ColumbBsn	47.468283	-119.67553	Washington	WA-CB
PIGLET_CB	ColumbBsn	47.470159	-119.69782	Washington	WA-CB
SULLIVAN	ColumbBsn	47.494451	-119.68468	Washington	WA-CB
TERRA	ColumbBsn	47.479282	-119.69323	Washington	WA-CB
TULIP	ColumbBsn	47.47496	-119.67769	Washington	WA-CB
8M1	IDNLemhi	44.243155	-112.99402	Idaho	IDN
8M7	IDNLemhi	44.243155	-112.9938	Idaho	IDN
ALLIE_ID	IDNINL	43.503162	-111.83267	Idaho	IDN
ALLIE_NV	NVParisCk	40.038041	-114.98734	Nevada	NV
ALLISON	NVOven	39.671453	-115.17126	Nevada	NV
CAS2	IDSCas	42.161292	-113.84039	Idaho	IDS
CG128	IDNLemhi	44.700721	-113.28742	Idaho	IDN
DSC13	IDNLemhi	44.720071	-113.51247	Idaho	IDN
JH01	IDNCamas	43.25305	-114.66389	Idaho	IDN
JH06	IDNCamas	43.25588	-114.66285	Idaho	IDN
MID162	IDNLemhi	44.720071	-113.51247	Idaho	IDN
MOR5	IDNCamas	43.261234	-114.80433	Idaho	IDN
MOR9	IDNCamas	43.260268	-114.80266	Idaho	IDN

OW41	IDSOwhyee	42.564107	-116.2367	Idaho	IDS
OW44	IDSOwhyee	42.563196	-116.23409	Idaho	IDS
OW45	IDSOwhyee	42.562015	-116.23376	Idaho	IDS
OW46	IDSOwhyee	42.561003	-116.20359	Idaho	IDS
RC466	IDNLemhi	44.663819	-113.22915	Idaho	IDN
RC467	IDNLemhi	44.661053	-113.22333	Idaho	IDN
RC472	IDNLemhi	44.665069	-113.22036	Idaho	IDN
RC477	IDNLemhi	44.666578	-113.22628	Idaho	IDN
SC478	IDNLemhi	44.701537	-113.33711	Idaho	IDN
SC481	IDNLemhi	44.700034	-113.33951	Idaho	IDN
TC3	IDNWSalm	45.111017	-113.94587	Idaho	IDW
TC4	IDNWSalm	45.110002	-113.94469	Idaho	IDW
TC5	IDNWSalm	45.118513	-113.99173	Idaho	IDW
TC6	IDNWSalm	45.114488	-113.94449	Idaho	IDW
W1924	IDNINL	43.503162	-111.83267	Idaho	IDN
W1927	IDNINL	43.503162	-111.83267	Idaho	IDN
W1928	IDNINL	43.503162	-111.83267	Idaho	IDN
W1931	IDNINL	43.503162	-111.83267	Idaho	IDN
WS21	IDNLemhi	44.644927	-113.25694	Idaho	IDN
WS26	IDNLemhi	44.61111	-113.25111	Idaho	IDN
WS27	IDNLemhi	44.642222	-113.25222	Idaho	IDN
WT10	IDNLemhi	44.49781	-113.94033	Idaho	IDN
WT5	IDNLemhi	44.41111	-113.94111	Idaho	IDN
WT8	IDNLemhi	44.42222	-113.94222	Idaho	IDN
WT9	IDNLemhi	44.43333	-113.94333	Idaho	IDN
BAD2	Montana	45.36013	-111.96633	Montana	MT
BAD4	Montana	45.31111	-111.96111	Montana	MT
BRADLEY	NVButteVly	39.658678	-115.16102	Nevada	NV
BSC10	Montana	44.829292	-113.0178	Montana	MT
BSC7	Montana	44.821111	-113.01111	Montana	MT
BSC8	Montana	44.822222	-113.02222	Montana	MT
BSC9	Montana	44.823333	-113.03333	Montana	MT
EC1	Montana	44.997686	-113.10982	Montana	MT
MH2	Montana	44.677269	-112.11052	Montana	MT
MH3	Montana	44.671111	-112.11111	Montana	MT
SAGE1	Montana	45.096475	-112.2701	Montana	MT
SAGE2	Montana	45.091111	-112.27111	Montana	MT
SAGE5	Montana	45.092222	-112.27222	Montana	MT
SAGE9	Montana	45.093333	-112.23333	Montana	MT
SW3	Montana	45.36013	-111.96633	Montana	MT
BUDDY	NVButteVly	39.658021	-115.16508	Nevada	NV

CHOWDER	NVSandMnd	39.854203	-115.14602	Nevada	NV
DUJOUR	NVButteVly	39.720366	-115.18396	Nevada	NV
FRICASE	NVButteVly	39.670375	-115.16064	Nevada	NV
HARRIET	NVParisCk	40.037323	-114.98695	Nevada	NV
INDIGOA	NVButteVly	39.687161	-115.17789	Nevada	NV
KJGB-B180	NVJigElk	40.442841	-115.98472	Nevada	NV
KWGA-B64	NVWGulch	38.030443	-114.37653	Nevada	NV
LISA	NVButteVly	39.082107	-114.77248	Nevada	NV
PEDRO	NVButteVly	39.671453	-115.17126	Nevada	NV
PIERRE	NVOven	39.081538	-114.77284	Nevada	NV
POPEYE	NVParisCk	40.028875	-114.98089	Nevada	NV
RAS	NVOven	39.084344	-114.7707	Nevada	NV
SOUP	NVButteVly	40.02907	-114.9812	Nevada	NV
FLINTI	Oregon	42.757143	-119.9842	Oregon	OR
FURAHA	Oregon	42.225468	-119.43128	Oregon	OR
SBFALE2	Oregon	42.225388	-119.43167	Oregon	OR
SWISS_MISS	Oregon	42.741692	-120.03723	Oregon	OR
BAYER	NUTRich	41.853109	-111.12653	Utah north	UTN
BOOM	NUTRich	41.84741	-111.12363	Utah north	UTN
GANDOFF	NUTRand	41.734101	-111.22086	Utah north	UTN
KANGA	NUTRand	41.732625	-111.21544	Utah north	UTN
PIGLET_UT	NUTRich	41.849653	-111.12427	Utah north	UTN
ROBIN	NUTRich	41.732325	-111.21715	Utah north	UTN
SUZI	NUT6mile	41.854154	-111.15582	Utah north	UTN
TONI	NUTRand	41.732209	-111.21639	Utah north	UTN
TUTTUT	NUTRand	41.930172	-111.20652	Utah north	UTN
BETTY	SUTOtterCk	38.208455	-111.9994	Utah south	UTS
BRONCO	SUTOtterCk	38.208455	-111.9994	Utah south	UTS
DEGI	SUTPrkrMt	38.208455	-111.9994	Utah south	UTS
EARLY_BIRD	SUTPrkrMt	38.208455	-111.9994	Utah south	UTS
HORK	SUTOtterCk	38.208455	-111.9994	Utah south	UTS
LOA	SUTPrkrMt	38.208455	-111.9994	Utah south	UTS
PEEPER	SUTOtterCk	38.208455	-111.9994	Utah south	UTS
SUKH2	SUTPrkrMt	38.208455	-111.9994	Utah south	UTS
TOLAI	SUTOtterCk	38.208455	-111.9994	Utah south	UTS
TULAROO	SUTPrkrMt	38.208455	-111.9994	Utah south	UTS
BEAUDREAUX	Wyoming	42.377352	-108.18847	Wyoming	WY
CADDIS	Wyoming	42.377352	-108.18847	Wyoming	WY
DIGGER	Wyoming	42.377352	-108.18847	Wyoming	WY
JUEL	Wyoming	42.377352	-108.18847	Wyoming	WY
MICK	Wyoming	42.377352	-108.18847	Wyoming	WY

SANDY	Wyoming	42.377352	-108.18847	Wyoming	WY
STUNNEL	Wyoming	42.377352	-108.18847	Wyoming	WY
UNO	Wyoming	42.377352	-108.18847	Wyoming	WY

Table S2. Tables representing a) F_{ST} values and b) Heterozygosity levels in pygmy rabbit samples genotyped at 9794 SNPs (complete set) with relatedness cutoff values of >0.33 and >0.40 for Washington samples. Central range (labeled here as Great Basin, GB) consists of California (CA, n=4), Nevada (NV, n=17), Oregon (OR, n=4), Idaho (ID, n=36), and Montana (MT, n=14). UTN-WY region consists of northern Utah (UTN, n=9) and Wyoming (WY, n=8). UTS represents southern Utah (UTS, n=10). Washington varies in samples size based on relatedness cutoff (WA-CB, n=16, 8, 21, respectively).

a)

Relatedness cutoff >0.33			
	GB	UTN-WY	UTS
UTN-WY	0.133		
UTS	0.158	0.281	
WA-CB	0.363	0.476	0.497

Relatedness cutoff >0.40			
	GB	UTN-WY	UTS
UTN-WY	0.133		
UTS	0.158	0.281	
WA-CB	0.383	0.505	0.524

No Relatedness Cutoff			
	GB	UTN-WY	UTS
UTN-WY	0.133		
UTS	0.158	0.281	
WA-CB	0.395	0.517	0.534

b)

Expected Heterozygosity				
	GB	UTN-WY	UTS	WA-CB
Related>0.33	0.2	0.18	0.18	0.13
Related>0.40	0.2	0.18	0.18	0.12
Complete	0.2	0.18	0.18	0.12

Observed Heterozygosity				
	GB	UTN-WY	UTS	WA-CB
Related>0.33	0.14	0.15	0.15	0.10
Related>0.40	0.14	0.15	0.15	0.10
Complete	0.14	0.15	0.15	0.10

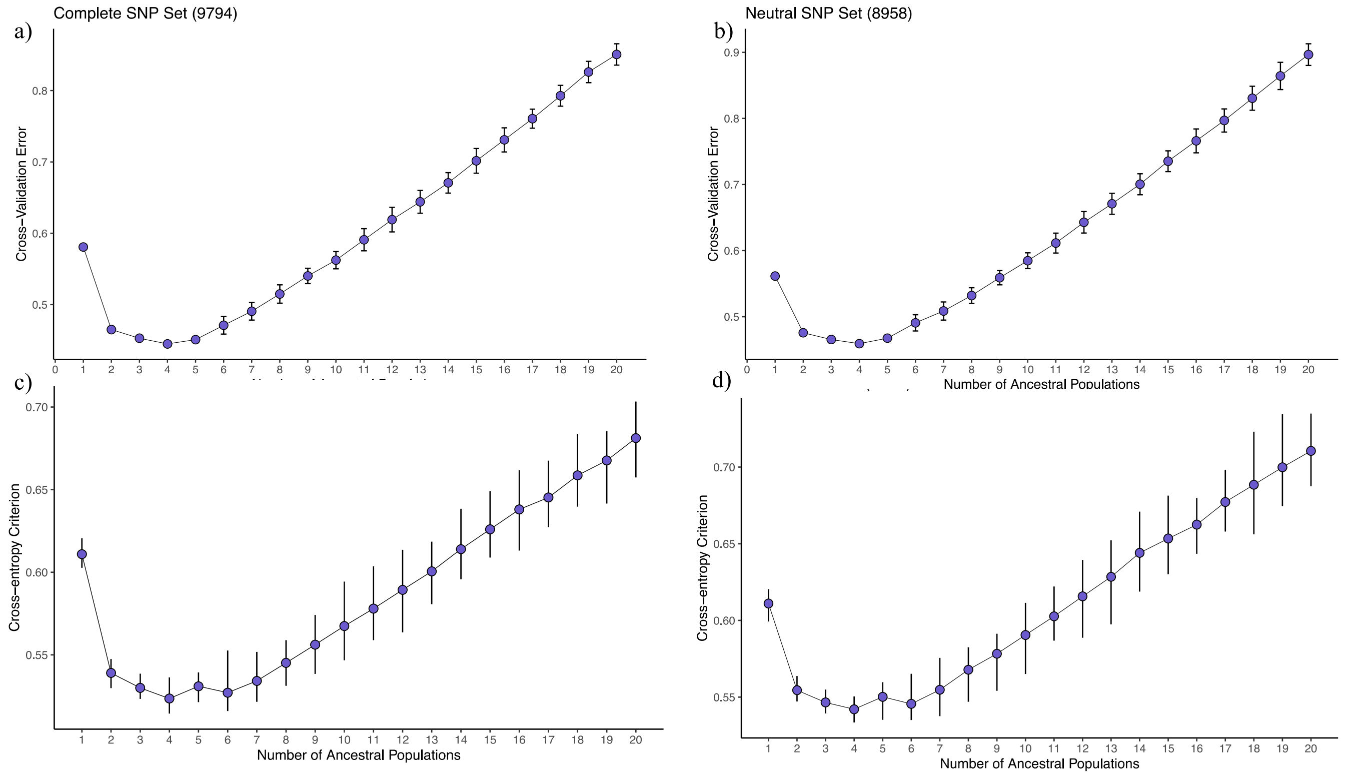

Figure S1. ADMIXTURE cross-validation error plots for K = 1-20 across 123 pygmy rabbit samples genotyped at a) 9794 SNPs (complete set) and b) 8958 SNPs (neutral set). Snmf cross-entropy plots for K = 1-20 across 123 samples genotyped with c) complete set and d) neutral set. 50 replicates are summarized for each value of K.

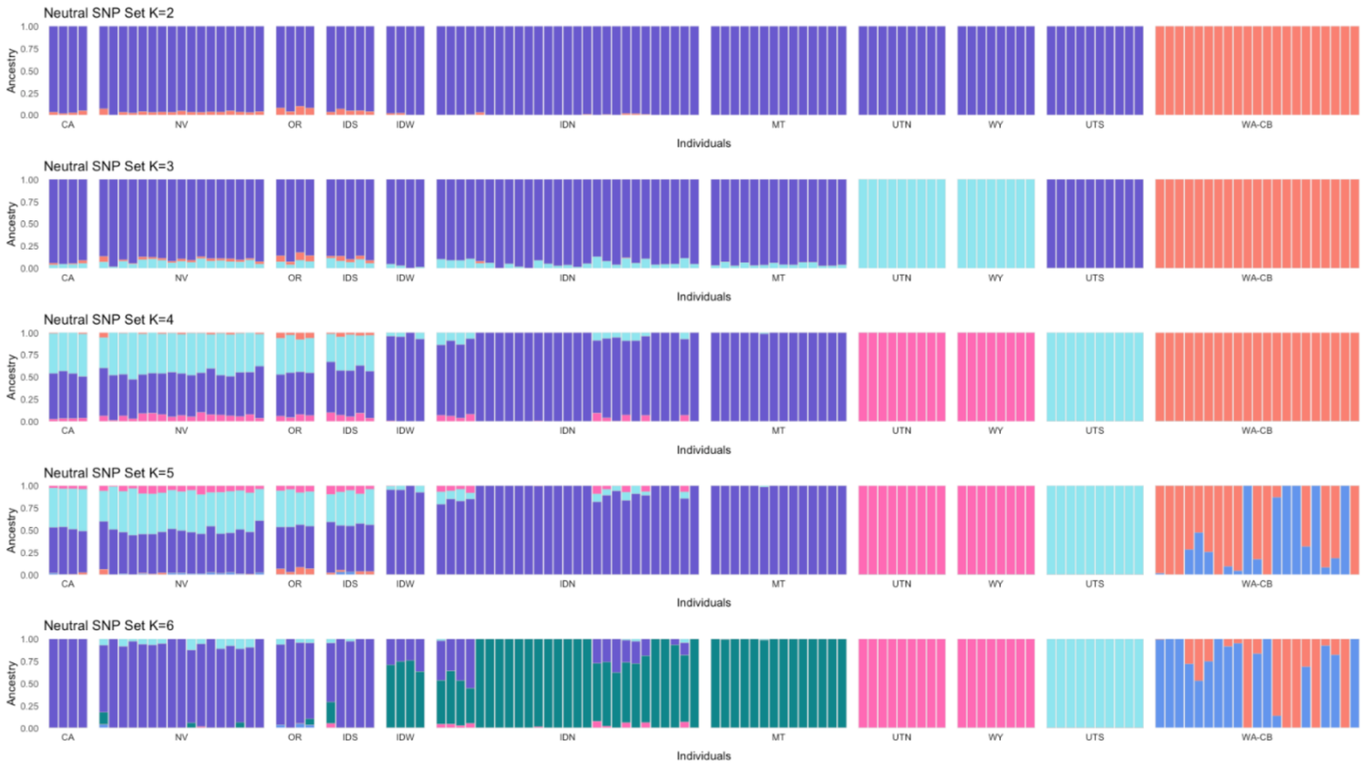

Figure S2. Graphical representation of ancestry assignments from ADMIXTURE for 123 pygmy rabbit samples genotyped at 8959 SNPs (neutral set) for K = 2-6. K = 4 is the optimal value based on lowest cross-validation values (Figure S1). Samples are oriented north-south within each region. Regions and samples sizes are California (CA, n=4), Washington (WA-CB, n=21), Nevada (NV, n=17), Oregon (OR, n=4), Idaho north of the Snake River (IDN, n=27), Idaho south of the Snake River (IDS, n=5), Idaho west of the Salmon River (IDW, n=4), northern Utah (UTN, n=9), southern Utah (UTS, n=10), Wyoming (WY, n=8), and Montana (MT, n=14).

a)

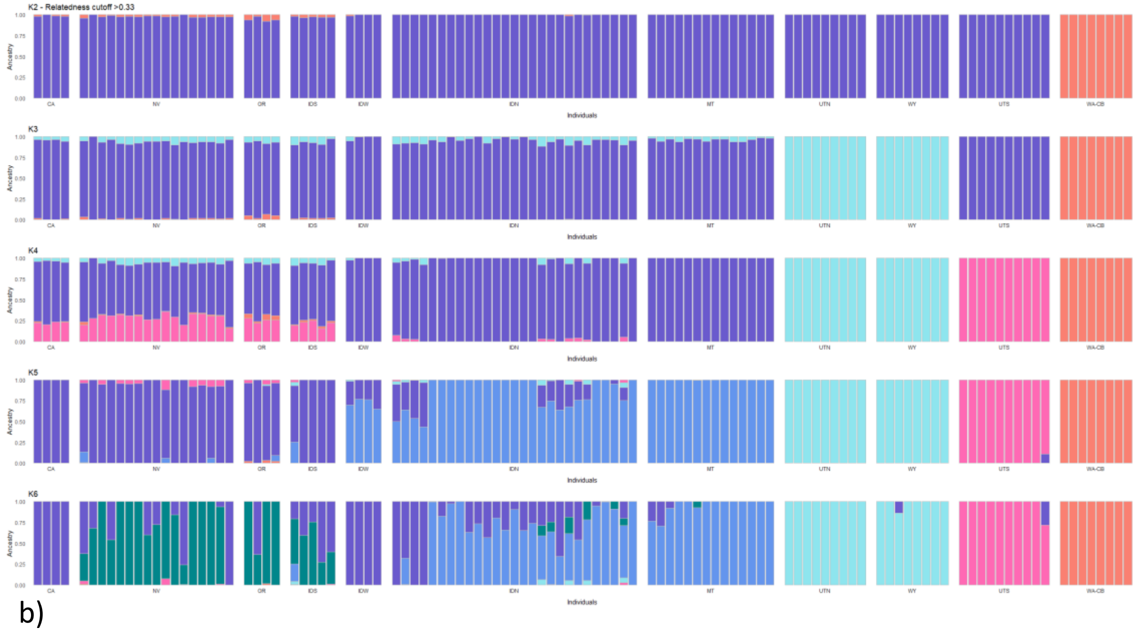

b)

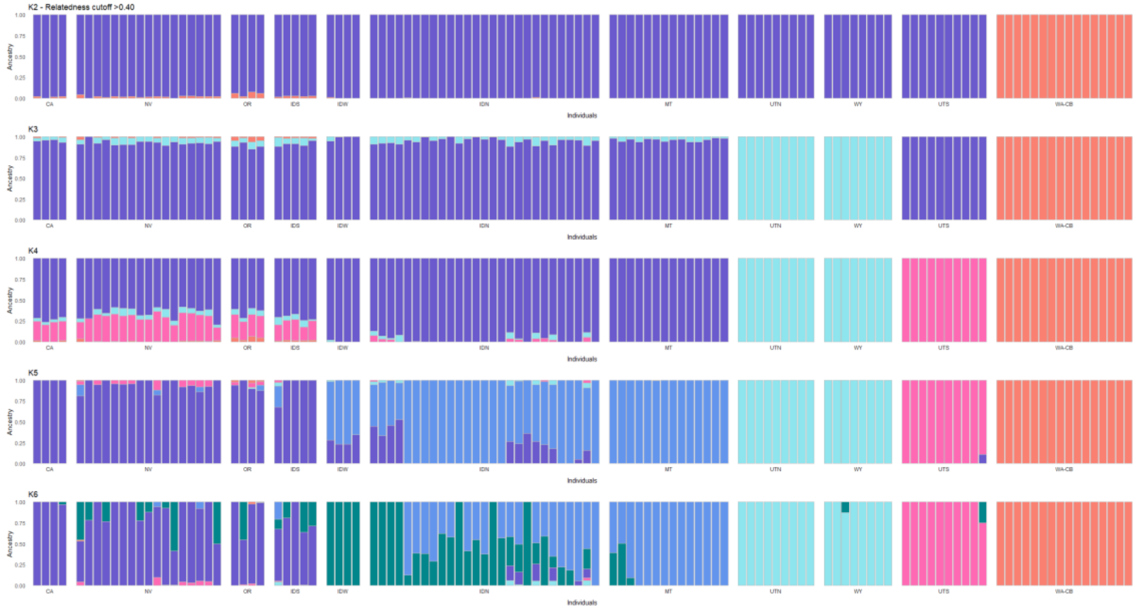

Figure S3. Graphical representation of ancestry assignments from ADMIXTURE for pygmy rabbit samples genotyped at 9794 SNPs (complete set) for K = 2-6 with relatedness cutoff values of a) >0.33 and b) >0.40 for Washington samples. K = 4 is the optimal value based on lowest cross-validation values (Figure S3). Samples are oriented north-south within each region. Regions and samples sizes are California (CA, n=4), Washington (WA-CB, n=8, 16, respectively), Nevada (NV, n=17), Oregon (OR, n=4), Idaho north of the Snake River (IDN, n=27), Idaho south of the Snake River (IDS, n=5), Idaho west of the Salmon River (IDW, n=4), northern Utah (UTN, n=9), southern Utah (UTS, n=10), Wyoming (WY, n=8), and Montana (MT, n=14).

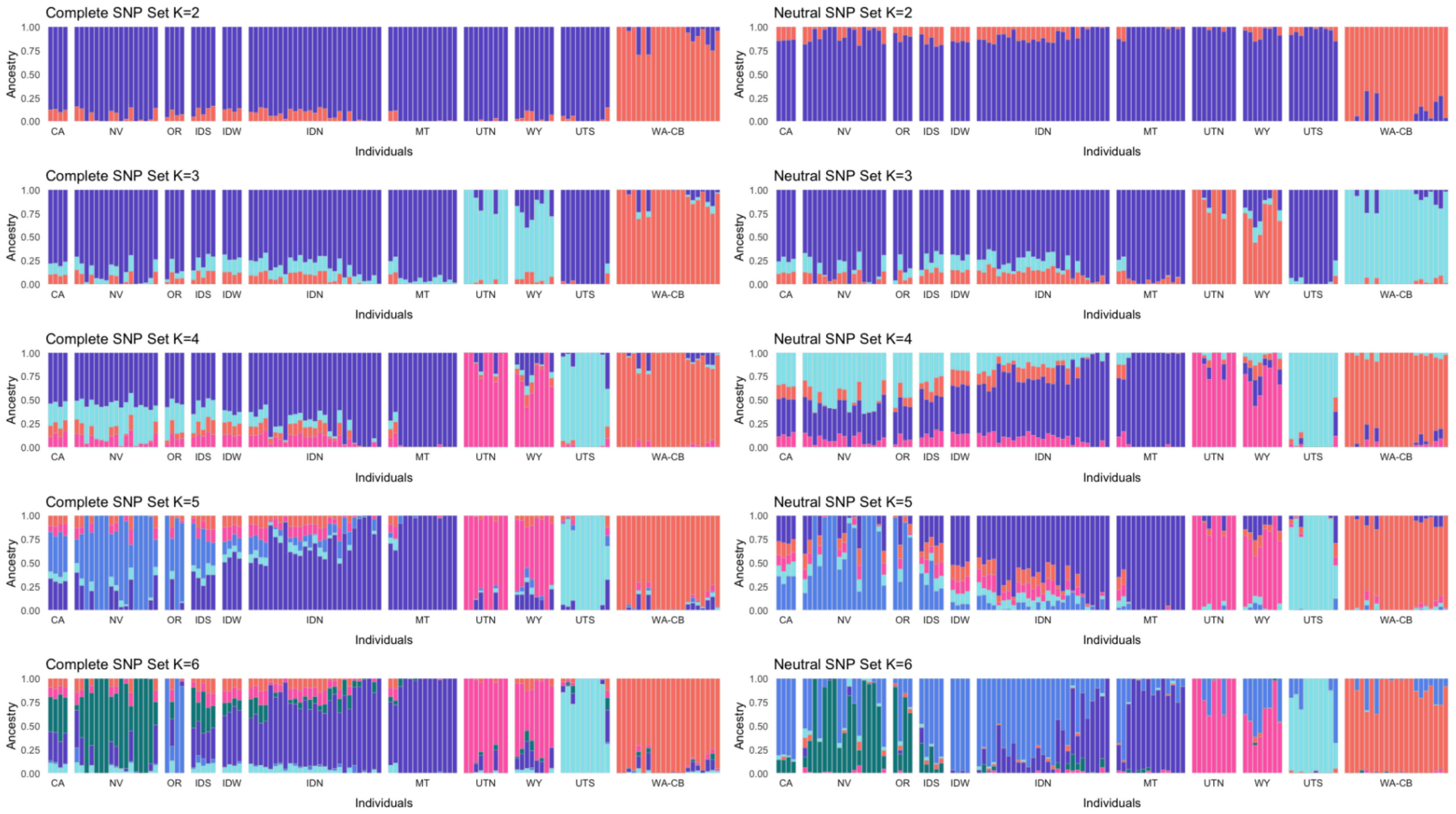

Figure S4. Graphical representation of ancestry assignments from *snmf* for 123 pygmy rabbit samples genotyped at 9794 SNPs (complete set) and 8959 SNPs (neutral set) for K = 2-6. K = 4 is the optimal value based on lowest cross-validation values (Figure 3c and 3d) but some support for K = 6. Samples are oriented north-south within each region. Regions and samples sizes are California (CA, n=4), Washington (WA-CB, n=21), Nevada (NV, n=17), Oregon (OR, n=4), Idaho north of the Snake River (IDN, n=27), Idaho south of the Snake River (IDS, n=5), Idaho west of the Salmon River (IDW, n=4), northern Utah (UTN, n=9), southern Utah (UTS, n=10), Wyoming (WY, n=8), and Montana (MT, n=14).

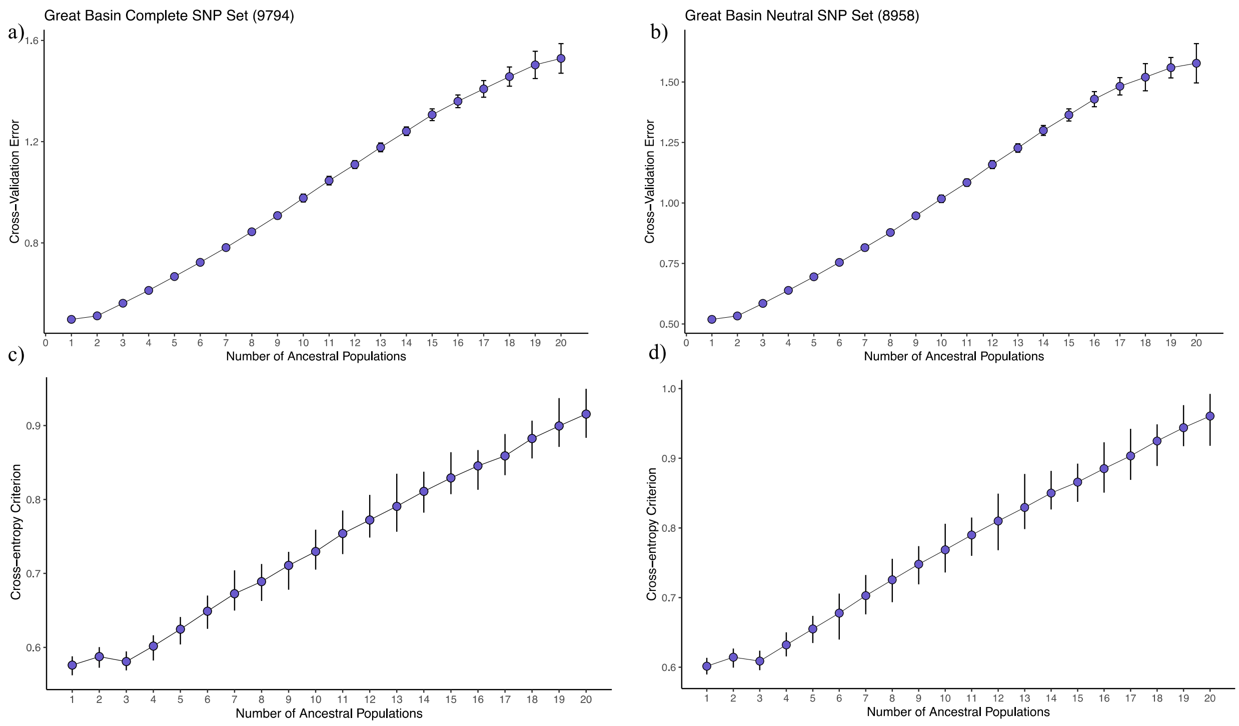

Figure S5. ADMIXTURE cross-validation error plots for 50 replicates of K = 1-20 across 75 pygmy rabbit samples from the central portion of the species range, genotyped at a) 9794 SNPs (complete set) and b) 8958 SNPs (neutral set). *Snmf* cross-entropy plots for K = 1-20 across 75 samples genotyped with c) complete set and d) neutral set.

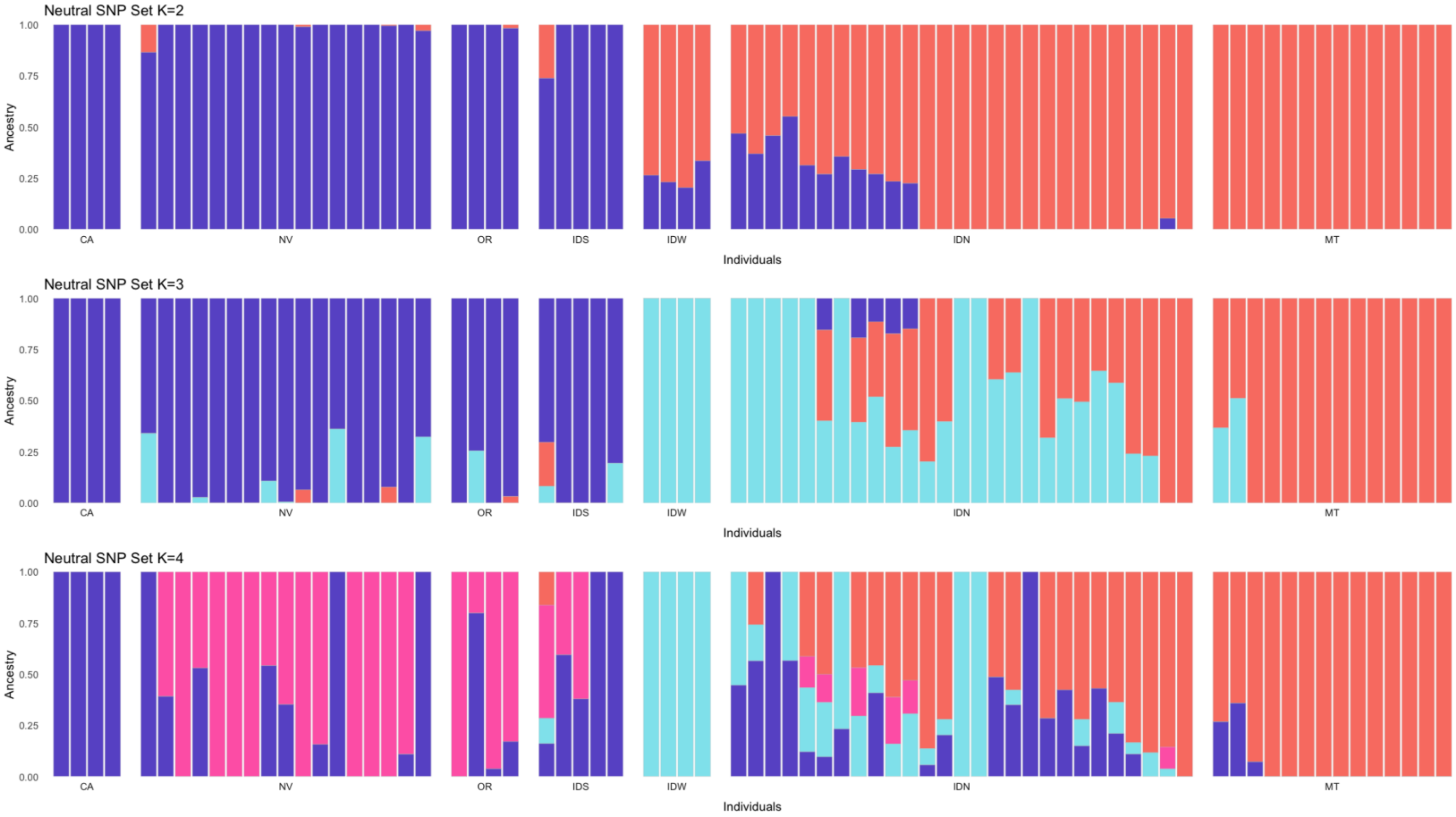

Figure S6. Graphical representation of ancestry assignments from ADMIXTURE for 75 central-range pygmy rabbit samples genotyped at 8959 SNPs (neutral set) for K = 2-4. Samples are oriented north-south within each region. Regions and samples sizes are California (CA, n=4), Nevada (NV, n=17), Oregon (OR, n=4), Idaho north of the Snake River (IDN, n=27), Idaho south of the Snake River (IDS, n=5), Idaho west of the Salmon River (IDW, n=4), and Montana (MT, n=14).

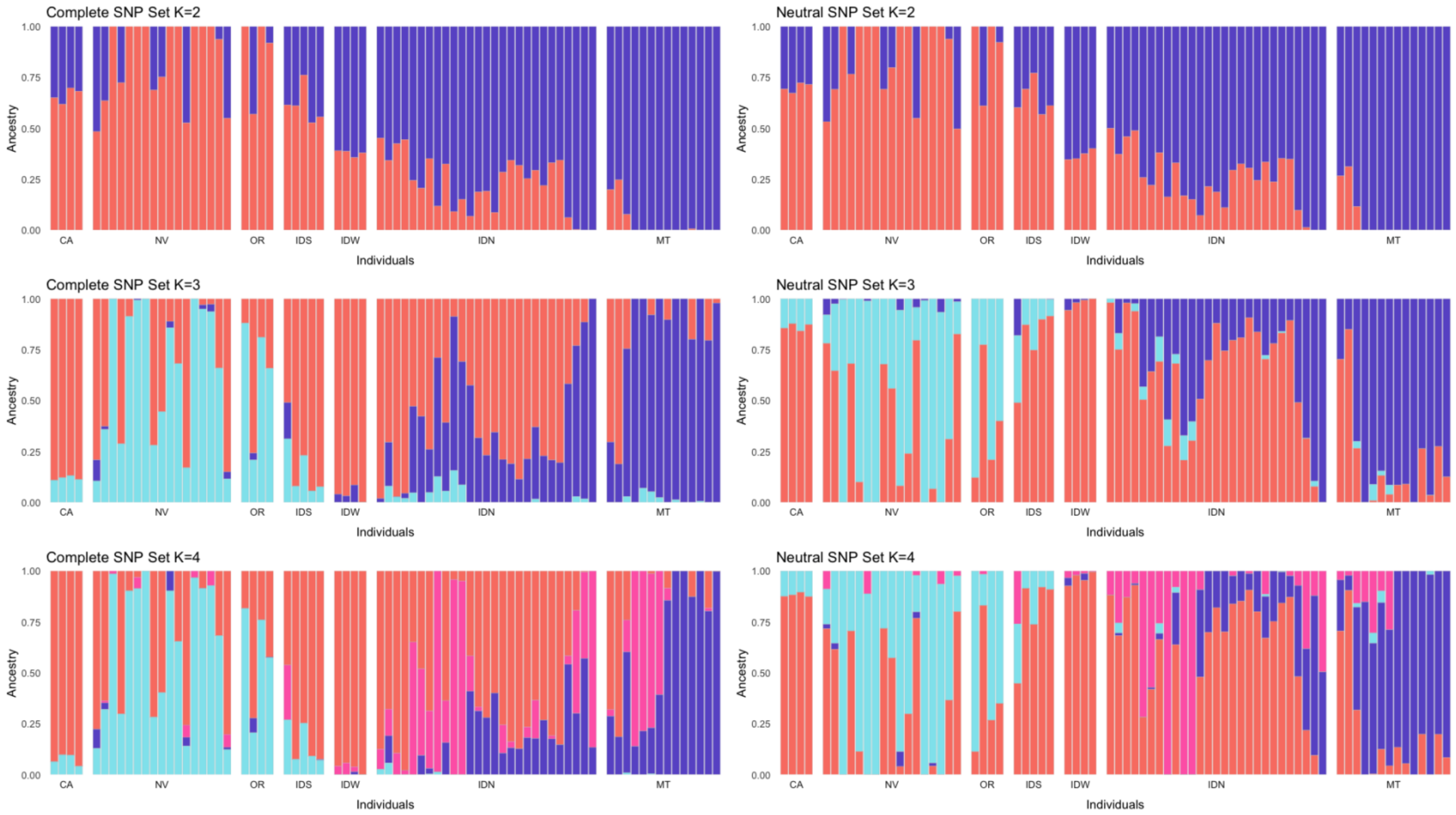

Figure S7. Graphical representation of ancestry assignments from *snmf* for 75 central-range pygmy rabbit samples genotyped at 9794 SNPs (complete set) and 8959 SNPs (neutral set) for K = 2-4. Samples are oriented north-south within each region. Regions and samples sizes are California (CA, n=4), Nevada (NV, n=17), Oregon (OR, n=4), Idaho north of the Snake River (IDN, n=27), Idaho south of the Snake River (IDS, n=5), Idaho west of the Salmon River (IDW, n=4), and Montana (MT, n=14).

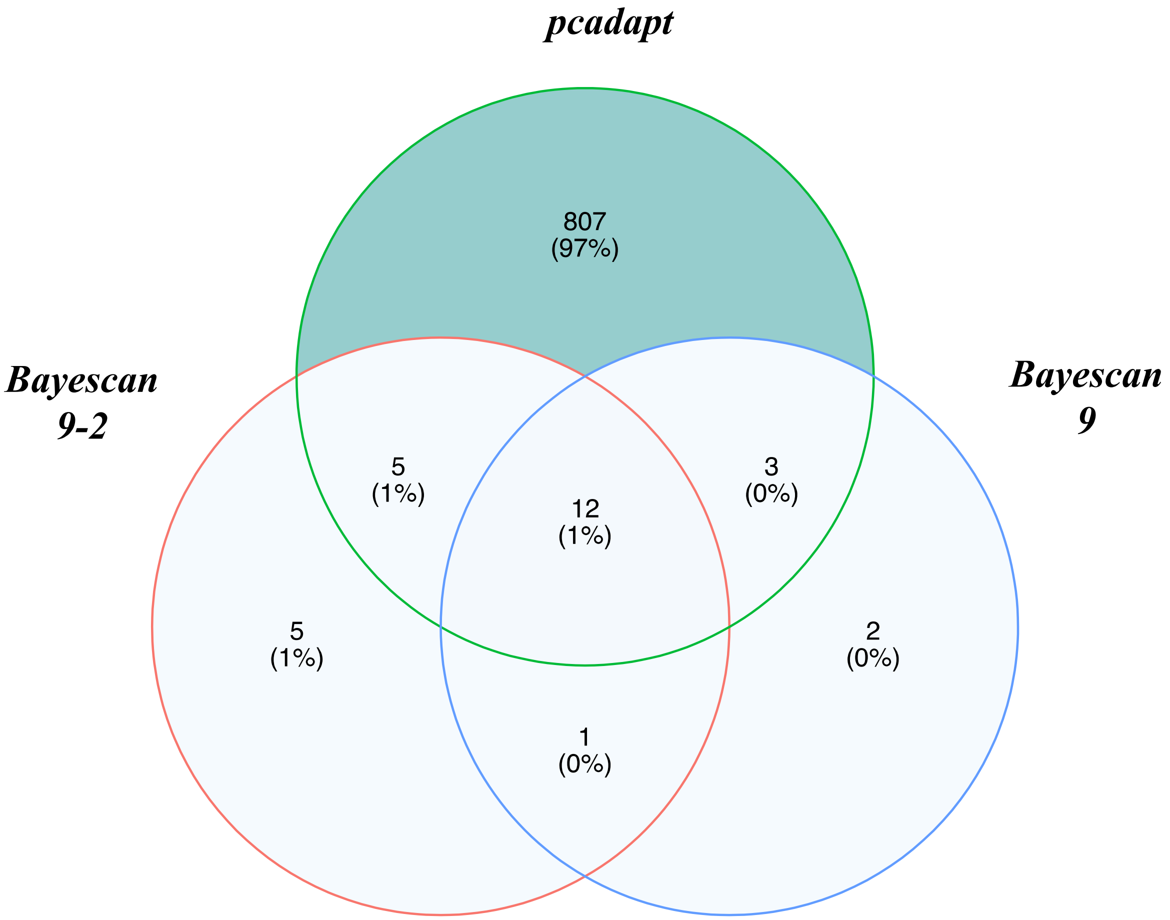

Figure S8. Venn Diagram representing the outlier loci identified with pcadapt and BayeScan on the complete pygmy rabbit data set consisting of 9794 SNPs across 123 samples. The BayeScan 9 version has populations assigned by region (Figure 1), and BayeScan 9-2 has population assigned by state.
